# What’s the TEE: Metrics of Temperature Extremes in Europe NUTS Regions (1980-2024)

**DOI:** 10.1038/s41597-025-05352-7

**Published:** 2025-07-01

**Authors:** Sara R. Ronnkvist, Zoé Haskell-Craig, Abbie Robinson, Risto Conte Keivabu, Mathew E. Hauer, Domenico Bovienzo, Emilio Zagheni

**Affiliations:** 1 University of Wisconsin - Madison, Sociology, Madison, 53711 USA; 2 Digital and Computational Demography Lab, Max Planck Institute for Demographic Research, Rostock, Germany; 3 New York University School of Global Public Health, Biostatistics, New York, 10003 USA; 4 The Pennsylvania State University, University Park, USA; 5 Florida State University, Sociology, Tallahassee, FL 32317 USA; 6 Cá Foscari University, Venice, Italy

**Keywords:** Atmospheric dynamics, Environmental health

## Abstract

We generate datasets quantifying extreme temperature exposure in Europe using a variety of metrics at two sub-national spatial scales (NUTS 2 and NUTS 3) and three temporal scales (daily, extreme temperature wave, and yearly) from 1980-2024. These datasets capture the breadth of temperature metrics used in epidemiology, demography and environmental literature with 67 different metrics: including regionally-unusual temperature events (defined as temperatures above/below the 95^*th*^/5^*th*^ percentile of historical temperatures) and periods of sustained (consecutive day) exposure to extreme temperatures. Although publicly available, climate data format and spatial resolution rarely matches the structure, scale, and extent used to disseminate government statistics on health, economic, and demographic variables, and manipulating raw data is computationally expensive. Here we provide temperature data in a user-friendly format which can easily be linked to EuroStat. Our open-sourced code and reproducible methods can be extended to produce similar datasets at the global scale.

## Background & Summary

Climate change has already altered and is expected to continue to impact the prevalence of extremely hot and cold days across the globe^1,2^. Extreme temperatures have multiple implications for population health^3^. For example, exposure to extreme heat is associated with increased heat stroke^4^, hospitalization^5^, and mortality^6^, while extreme cold is associated with increased risk of hypothermia, respiratory illnesses and mortality^7^.

Despite the clear importance of extreme temperatures on population health, the lack of easy-to-use temperature data acts as a barrier to research on climate impacts^8–12^. Data from ground-based stations is challenging to work with: either available only from commercial sources (e.g. The Weather Network, Weather Underground) or requiring harmonization across different government datasets where temporal period, resolution, data format, and metadata language vary by country (e.g. MeteoNet from METEOFRANCE, the Climate Data Center of the Deutscher Wetterdienst). While satellite-derived, high resolution temperature data are often freely available, these are typically stored at very fine spatial and temporal resolutions which require intensive computing resources and geospatial programming knowledge to process. In particular, Soranno *et al*. (2015) note investigators must go through a complicated process to access data in various formats, often of different spatio-temporal resolutions, and in disparate storage systems. These factors make certain temperature datasets unfeasible for non-specialist use, who might lack adequate training or access to high throughput computing. Myroshnychenko *et al*. (2015) further emphasized the previously described barriers to data usage, calling for improved environmental dataset accessibility to ensure that researchers and policymakers have access to important data that informs research and mitigation planning. Other limitations, such as the lack of spatial coverage in common spatial units, additionally hinders research. The unmet needs of public access and spatial coverage of Europe further motivates the need for a user-friendly environmental dataset for research purposes. Improving access to temperature data at the same spatial resolution used to publish health, economic, and state/government infrastructure data will facilitate research furthering our understanding of temperature-related impacts on population processes.

Furthermore, multiple metrics of extreme temperature are common in the literature, complicating analyses and comparisons between studies. Some studies use global thresholds to denote extreme temperatures (e.g.^13,14^), while others use localized metrics of extreme temperatures that situate extremes within a place’s climate (e.g.^15–17^). Here and throughout this paper we use the term ‘global’ metric to refer to a definition of extreme temperature that utilizes on a single temperature threshold for all regions (e.g. region A is considered to have experienced extreme heat on a date if the daily mean temperature is greater than 30 °C), whereas ‘local’ measures assign a different threshold for each region based on historical temperature trends (e.g. region A’s daily mean temperature is greater than the 90^*th*^ percentile of historical daily temperatures in region A). Additionally, metrics of extreme temperatures also vary across temporal scale. For example, previous studies have used total number of days above a threshold in a year, heatwaves in a year, or total number of days in a year that were in a heatwave to define extreme heat exposure.

**Fig. 1:**
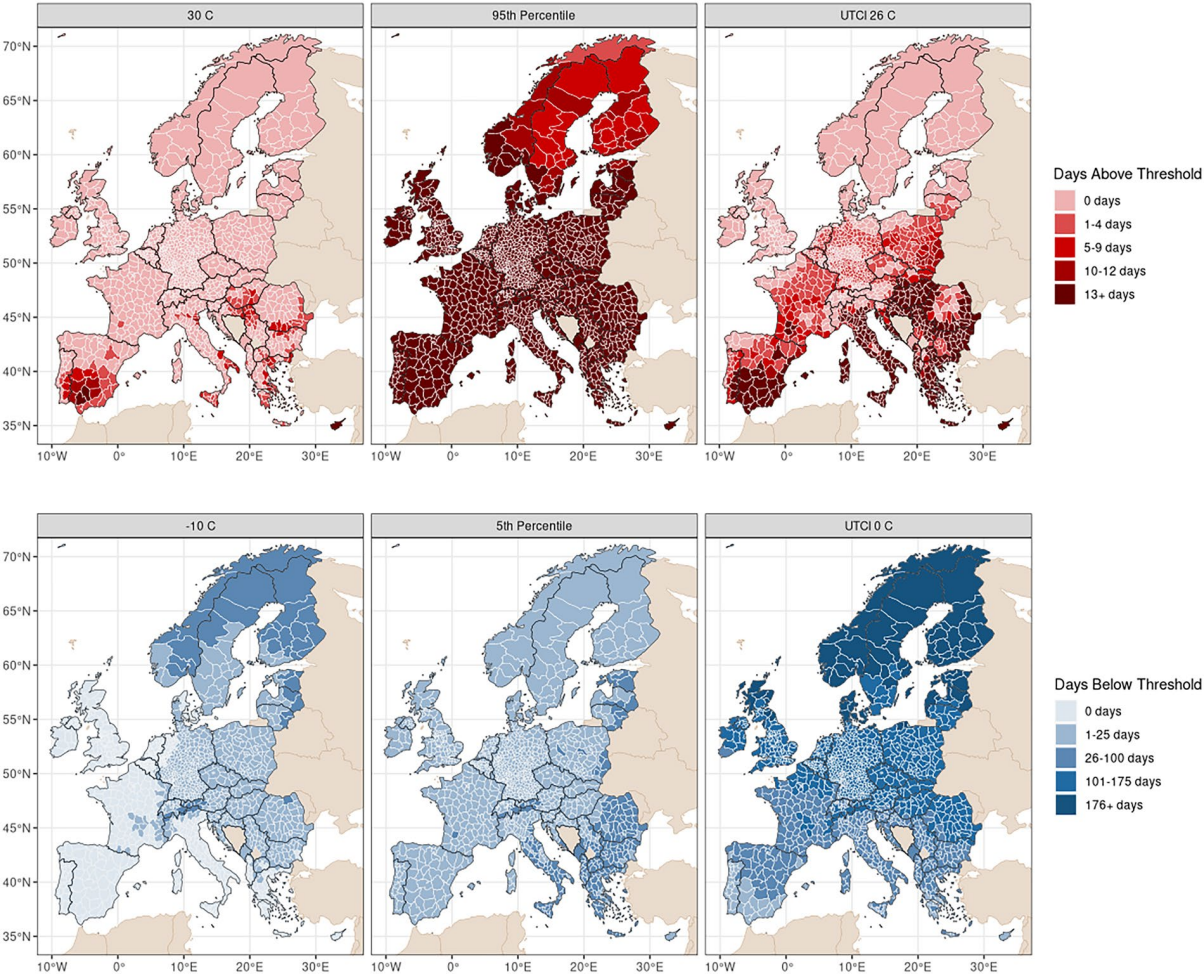
Spatial patterning of extreme temperatures across Europe in 2012. Note that the regions identified as experiencing a high number of extreme days varies substantially based on the metric used. Southern regions experienced the highest number of days over 30 °C while days below  −10 °C occurred predominantly in Northeastern Europe. Northern Norway, Sweden, and Finland experienced a cooler summer with less than 12 days above the historical 95^*th*^ percentile. Universal Thermal Climate Index (UTCI) metrics include wind and humidity. Continuous extreme temperature measures are binned following natural breaks in the distribution.

To make studying the impacts of extreme temperature more feasible, we produce a series of temperature datasets for Europe (EU). Europe has experienced a large increase in temperature-related mortality in the past 20 years^6^, and due to an aging population, is expected to be particularly vulnerable to extreme temperatures in the future^18^. Here, we provide six datasets comprising of two spatial scales (NUTS 2, NUTS 3) and three temporal scales (daily, yearly, wave) of temperature for Europe from January 1st, 1980 to December 31st, 2024. The datasets include temperature estimates (i.e. measures and metrics). In this paper, measures refer to recorded values of temperature and metrics summarize the severity of a temperature exposure period. More precisely, we provide daily averages for the measures of temperature and Universal Thermal Climate Index (UTCI) and yearly totals of days above/below absolute and relative temperatures and extreme temperature wave data (i.e. as waves of consecutive days) at the NUTS 2 and NUTS 3 level. These spatial and temporal scales are consistent with publicly available administrative demographic data. Notably, the spatial patterning of extreme temperatures varies substantively across the different metrics. To illustrate this, we mapped selected yearly metrics of extreme heat and cold in 2012 in Fig. 1. For graphical clarity, we converted continuous measures of extreme temperature to categorical variables using natural breaks in the distribution as cutoffs for non-zero extreme temperature exposure. Users should consider how extreme temperatures are related to their outcome of interest when selecting which metric to use.

Additionally, we provide the underlying computer code that could be readily adapted to create temperature measures for other global regions and/or for researchers to create their own temperature metrics. In the following sections, we describe how we constructed each data file and subsequent temperature metrics.

## Methods

**Fig. 2:**
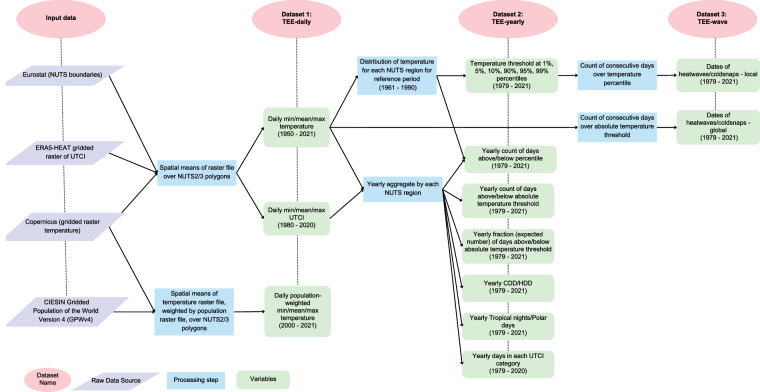
Analysis pipeline displaying raw data used to produce each final Temperature Extremes in Europe (TEE) dataset. Daily measures (TEE-daily) for each NUTS region are developed from publicly available raw data. These are aggregated to produce six yearly metrics (TEE-yearly). Consecutive days of extreme temperature (TEE-waves) are computed using both absolute and relative thresholds. UTCI stands for Universal Thermal Climate Index, CDD stands for Cooling Degree Days, and HDD stands for Heating Degree Days.

This section is organized into three main subsections that describe how we constructed temperature metrics at each of the three temporal scales - daily, yearly, and wave. Figure 2 details the data analysis pipeline. We first construct the TEE-daily dataset containing daily measures of temperature across each region. We then aggregate these daily measures to produce six yearly metrics of extreme temperature exposure, compiled as the TEE-yearly dataset. We also identify the dates of consecutive days of extreme temperatures in the TEE-wave dataset. Table 1 provides a brief summary of these datasets.

**Table 1:** Overview of the three datasets. Variable names and definitions are listed in the codebook available online.

Dataset Name	Spatial Scale	Temporal Scale
TEE-daily	NUTS 2, NUTS 3	Day (by date)
TEE-yearly	NUTS 2, NUTS 3	Year
TEE-wave	NUTS 2, NUTS 3	Period (by date)

All three datasets are available at two spatial scales - NUTS 2 and NUTS 3. Nomenclature of territorial units for statistics (NUTS) regions are areas constructed by the European Union for statistical and policy purposes. NUTS 2 regions are basic or regional spatial units which are comparable to U.S. states. NUTS 3 units are nested within NUTS 2 regions and are comparable to U.S. counties. To identify NUTS regions, we used the 2021 NUTS shapefiles produced by EuroStat^19^. The statistical office of the European Union publishes EuroStat data, providing statistics, geographic boundary files, and other reports for the EU. To reduce download and processing time for users, we provide separate versions of each temporal dataset for NUTS 2 and NUTS 3 regions. We structure each dataset similarly and use the same variable names across datasets so users who wish to use multiple temporal or spatial scales may easily merge multiple datasets.

### TEE-daily: Raster Processing for Daily Temperature

We develop daily measures of temperature by converting raw climate data from gridded raster files to spatial means across each NUTS 2 and NUTS 3 region. We utilize the fifth generation of European ReAnalysis (ERA-5) Land hourly gridded climate data at 0.1 degree resolution for the period 1950-2024 for temperature^20^ and at a resolution of 0.25 degree for the period 1980-2024 for UTCI^21^. As a parameter of thermal comfort, UTCI is used to estimate human body coping response to heat stress and is often used in bioclimatic research^22–26^. Differing from other ambient air temperature datasets (e.g Climate Research Unit, European-Observational Baseline Surface), UTCI incorporates measures of air temperature, humidity, wind, and radiation to better represent the temperature an individual experiences. The ERA5-Land data is largely used in the existing literature and shows high accuracy in temperature measurement^27^. These data were originally created to validate climate models as a part of the EU ENSEMBLES project, but are now widely used to measure temperature extremes^28–30^.Also, these have shown to replicate well the exposure-response relationships observed in epidemiological studies when using ground truth data based on weather stations. Therefore, denoting that the resolution provided allows for rigorous analysis when averaged across large spatial units^31^. Nevertheless, the ERA-5 data is less accurate in providing information on highly localized phenomena such as heat islands, yet share similar temporal coverage to other existing datasets (e.g. E-OBS). All climate variables were retrieved from the Copernicus Data Store using the CDS API Python package for the spatial extent of Europe. While ERA5-Land is more widely used by researchers, UTCI may be a more useful measurement of temperature for studies focused on temperature related health outcomes. We include both measures to accommodate the data needs of a wider audience.

Raw climate data used to construct all measures are available from the Copernicus Data Store at https://cds.climate.copernicus.eu/. The fifth generation of European ReAnalysis (ERA-5) Land hourly gridded temperature data at 0.1 degree resolution can be downloaded at https://cds.climate.copernicus.eu/datasets/reanalysis-era5-land?tab=overviewand hourly gridded data for UTCI at 0.25 degree resolution can be downloaded at https://cds.climate.copernicus.eu/datasets/derived-utci-historical?tab=overview. Shapefile polygons delineating the European NUTS regions are available from EuroStat at https://ec.europa.eu/eurostat/web/gisco/geodata/statistical-units/territorial-units-statistics. Gridded population estimates were produced by the Center For International Earth Science Information Network and are available at 10.7927/H4JW8BX5.

We produce daily measures for each variable by taking the spatial mean of the raster grid cell values falling across the NUTS region. We weight all cells equally. For both temperature and UTCI, daily measures of the minimum, maximum, and mean values were based on the lowest, highest, and the average of hourly values for the region on the specific day (defined as the calendar date).

We also create population weighted measures of daily minimum, maximum, and mean temperature from 2000 - 2024 using population raster data at a resolution of 0.008 degrees from the Center for International Earth Science Information Network’s (CIESIN) Gridded Population of the World, Version 4^32^. In this case, temperature data of raster cells overlapping a given NUTS region are weighted by the corresponding population raster value, before a mean is taken. As CIESIN provides population estimates at 5-year intervals, we use data from the previous, most recent year when taking the weighted mean (e.g. for 2004 we use population data from 2000; for 2006 we use population data from 2005). We use the most conservative population weighting approach which does not assign future population densities to previous years. Assigning future population densities to previous years is problematic if a place experienced rapid population change within a short period of time.

These temperature measures are used to produce all subsequent metrics of extreme temperature. Additionally, they can be used by the user to create other metrics which may be better suited to their study.

### TEE-yearly: Computing Yearly Extreme Temperature Metrics

**Fig. 3:**
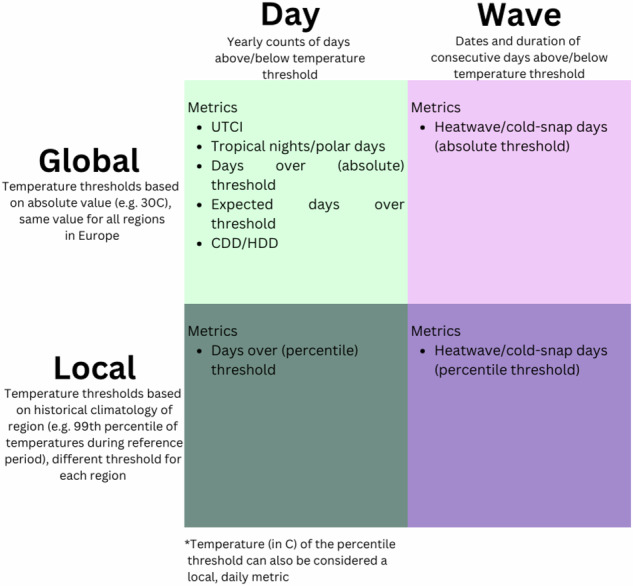
Extreme temperature metrics included in dataset, categorized by whether the measure is global (absolute thresholds) or local (relative thresholds), and computes days or waves. UTCI stands for Universal Thermal Climate Index, CDD stands for Cooling Degree Days, and HDD stands for Heating Degree Days.

Various definitions of extreme exposure have been used in the epidemiology, environmental demography, and climate-change literature. Here we strive to provide a variety of metrics. We create an important distinction between ‘global’ or ‘absolute’ metrics - which define extreme temperature as exceeding a set threshold, e.g. 30 °C - and ‘local’ or ‘relative’ metrics that define temperature extreme relative to the current or historical climate of that region (Fig. 3). As discussed above, global measures have the same cutoff temperature values across all spatial units while local measures have different cutoff values for every spatial unit. Another axis along which to categorize temperature metrics is whether they focus on single-day temperature extremes, or consider cumulative/consecutive days of exposure such as heatwaves or coldsnaps. In this dataset, we provide six metrics used in previous research studies. Table 2 provides an overview of these yearly metrics and the following sections describe how each metric is defined and calculated. In the following subsection, we describe how we computed each yearly temperature metric.

**Table 2:** Metric name and equation specifying how each metric is calculated.

Metric	Extreme	Equation	Temperature Measure	Units	Global/ Local
Tropical nights	Heat	${Y}_{{\rm{tropicalnights}}}={\sum }_{i=1}^{n}{I}_{i}({T}_{min} > 20)$	*T*_*min*_	Days	Global
Polar days	Cold	${Y}_{{\rm{polardays}}}={\sum }_{i=1}^{n}{I}_{i}({T}_{max} < 10)$	*T*_*max*_	Days	Global
Days above threshold	Heat	${Y}_{{\rm{above}}}(t)={\sum }_{i=1}^{n}{I}_{i}({T}_{mean} > t)$	*T*_*mean*_	Days	Global
Days below threshold	Cold	${Y}_{{\rm{below}}}(t)={\sum }_{i=1}^{n}{I}_{i}({T}_{mean} < t)$	*T*_*mean*_	Days	Global
Expect days above threshold	Heat	${Y}_{E[day > t]}(t)=1-{F}_{n}(t)=1-\frac{1}{n}{\sum }_{i=1}^{n}{I}_{i}({T}_{mean} < t)$	*T*_*mean*_	Unitless	Global
Expect days below threshold	Cold	${Y}_{E[day < t]}(t)={F}_{n}(t)=\frac{1}{n}{\sum }_{i=1}^{n}{I}_{i}({T}_{mean} < t)$	*T*_*mean*_	Unitless	Global
Cooling degree days	Heat	${Y}_{CDD}={\sum }_{i=1}^{n}(\frac{{T}_{max}-{T}_{min}}{2}-24){I}_{i}(\frac{{T}_{max}-{T}_{min}}{2} > 24)$	*T*_*max*_, *T*_*min*_	Degrees (°C)	Global
Heating degree days	Cold	${Y}_{HDD}={\sum }_{i=1}^{n}(20-\frac{{T}_{max}-{T}_{min}}{2}){I}_{i}(\frac{{T}_{max}-{T}_{min}}{2} < 20)$	*T*_*max*_, *T*_*min*_	Degrees (°C)	Global
Days above percentile	Heat	${Y}_{{\rm{above}}{\rm{p}}}(p)={\sum }_{i=1}^{n}{I}_{i}({T}_{max} > t(p,j))$	*T*_*max*_	Days	Local
Days below percentile	Cold	${Y}_{{\rm{below}}{\rm{p}}}(p)={\sum }_{i=1}^{n}{I}_{i}({T}_{max} < t(p,j))$	*T*_*max*_	Days	Local
Temperature percentile	Heat/Cold	Temperature at percentile *p*	*T*_*max*_	Degrees (°C)	Local

Note that most, but not all, metrics use daily mean temperature. Metrics are classified as global (defined based on an absolute threshold) or local (threshold based on historic temperatures in that region). When metrics are defined by a cut-off threshold we include a range of values (e.g. 1^*st*^,  5^*th*^,  10^*th*^ percentile).

#### Tropical nights/polar days

Tropical nights are defined as days in which the minimum temperature (*T*_*min*_) is above 20 °C. Polar days are days in which the maximum temperature (*T*_*max*_) is below 10 °C. This metric is commonly used in population-environment research to investigate various demographic related concepts (e.g., mortality, urbanization)^33–37^.

#### Days above/below absolute threshold

We compute the number of days with a mean temperature (*T*_*mean*_) above thresholds of *t* =  20 °C, 25 °C, 30 °C, 35 °C, 40 °C and 45 °C and below thresholds of *t* = −20 °C,  −15 °C, −10 °C,  −5 °C, 0 °C, and 5 °C. Absolute threshold is a metric that is widely used in the climate econometrics literature^3,38^.

#### Expected days above/below absolute threshold

For each threshold *t* =   −20 °C,  −15 °C,  −10 °C,  −5 °C, 0 °C, and 5 °C we find the expected number of days below the threshold in a given year by applying an empirical distribution function, *F*_*n*_. *F*_*n*_(*t*) is the fraction of observations less than or equal to *t*. For thresholds of *t* =  20 °C, 25 °C, 30 °C, 35 °C, 40 °C and 45 °C we compute the expected number of days above the threshold by taking 1 − *F*_*n*_(*t*).

#### Cooling degree days/heating degree days

Cooling degree days (CDD) calculate the number of degrees each daily temperature is above a temperature threshold of 24 °C^39^, considered to be the temperature at which individuals do not need a cooling mechanism (e.g. air conditioning) to feel comfortable. Likewise, heating degree days (HDD) sum the degrees below a threshold of 20 °C^40^. At this threshold, households need a heating mechanism to feel comfortable. Such metrics are often used to understand the energy demands of specific territories in terms of cooling and heating technologies for buildings^41–43^.

#### Temperature percentiles and days above/below percentile

For each location (NUTS 2 and NUTS 3 regions) we use historical temperature data from the years 1950 − 1990, as in previous similar studies^44,45^ to compute the 1^*st*^, 5^*th*^, 10^*th*^ and 90^*th*^, 95^*th*^ and 99^*th*^ percentiles for the mean daily temperature *T*_*mean*_. Previous research varies widely in the reference period used to produce percentiles, but periods generally cover at least 30 years and include dates from the 1980s^16,17,46^. Researchers wishing to use a different timeframe can re-create these metrics from our code by changing the reference period dates.

We report the temperature of these percentiles (in °C) as an additional local metric (note the temperature at these percentiles vary by region ID but not year). Given the temperature *t*(*p*, *j*) at each percentile *p* for each region *j*, we compute the number of days above (*p* = 0.90, 0.95, 0.99) and below (*p* = 0.01, 0.05, 0.1). These data define extreme temperature events and have been used to assess the relationship between heat variability and associated health risks^5,47,48^.

### TEE-wave: Heatwave/cold-snap dates

Research has also demonstrated the importance of sustained exposure to extreme temperatures, including the impact of extended periods of high and low temperatures on mortality^49–55^. In this dataset, we identify the dates of consecutive days (‘waves’) of extreme temperature for each region at the NUTS 2 and NUTS 3 level, as well as the length of each wave.

**Fig. 4:**
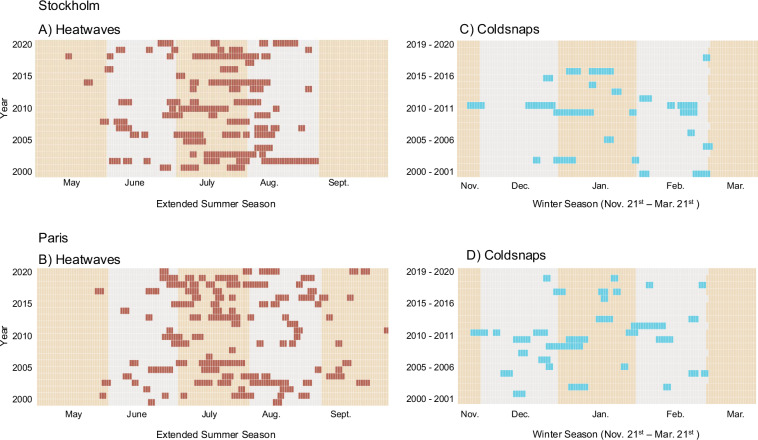
Dates of extreme temperature waves, defined as 3 or more consecutive days above/below the 95^*th*^/5^*th*^ percentile of historic temperatures for Stockholm, Sweden and Paris, France. Heatwaves in Paris extend well into September but are predominantly limited to June - August in Stockholm. Both Paris and Stockholm experienced a prolonged and early coldsnap in the winter of 2010 - 2011.

We construct extreme heat and cold waves using both global and local metrics of daily temperature. Specifically, we generate a start date and end date for heat waves above 20 °C, 30 °C, 40 °C, 90th percentile, 95th percentile, and the 99th percentile. Cold waves are denoted by days below 10 °C, 0 °C, −10 °C, 10th percentile, 5th percentile, and 1st percentile. A period where at least two days are above (or below) a given threshold is considered an extreme temperature wave in this dataset. We selected the lowest possible number of consecutive days for a wave to give users the greatest number of possible wave durations. The number of days in each wave is included as a column in the dataset, allowing users to filter to events of a specific length. For instance, users can use this dataset to identify the dates of heatwaves and coldsnaps (defined as >3 consecutive days of extreme temperature above/below the 95^*th*^/5^*th*^ percentile) occurring in two major European cities (Fig. 4) and match timing and duration of these events to relevant outcomes in their field of research.

## Data Records

Our final datasets and associated codebooks can be downloaded from the TEE-dataset data repository on Figshare^56^. All datasets are saved as .csv files and follow a consistent naming schema (dataset_spatiallevel.csv). For example, teewave_nuts2.csv is the TEE-wave dataset at the NUTS 2 level.

## Technical Validation

We use high quality raster data provided by Copernicus to construct measures and metrics of extreme temperatures. The high-accuracy of this data source has been established^20^ and these data are widely used in climate-health research^15,21,57,58^. To ensure no errors were made during raster processing we spot checked daily mean temperature for three NUTS 3 regions (corresponding to the cities of Paris, Helsinki, and Cagliari) against historical data from a commercial weather station website (https://www.wunderground.com/). For each location we randomly selected three dates during the time-period in which commercial records are available and matched these to daily mean temperature TEE-daily. We also matched dates for consecutive days and years to ensure that raster layers did not get offset. More comprehensive evaluation of the accuracy of satellite-based temperature datasets compared to ground-based stations is outside the scope of this paper (see for example^59^).

We performed a rigorous code review of scripts used to create yearly metrics (TEE-yearly) and heatwaves/coldsnaps (TEE-wave) with team members not involved in the initial code creation. We assessed data summary statistics to ascertain that there are no unreasonable values in the dataset (e.g. heatwaves lasting more than 300 days). At the time of writing, we are unaware of similar temperature datasets which would be the most appropriate comparison.

## Usage Notes

### Replicating Code for Other Regions or Metrics

While we only publish data for Europe, our code can be easily adapted to compute temperature extremes for other regions or at different spatial scales. One caveat is that users must have access to high throughput computing to process raster data. Since we used global raster data, the primary alteration to our code is changing the shapefile and associated variables (e.g. spatial unit identifier) used to compute spatial means. Other minor revisions to the code include changing the names of output files and variable names. Users may also wish to use a different measure of daily temperature to construct yearly temperature metrics. Since we generated daily measures of the minimum, mean, and maximum temperature, users can simply switch the temperature variable used in the metric construction.

## Data Availability

Code is available through a Github repository titled TEE-dataset (https://github.com/haskellcraigz/TEE-dataset/tree/main). To run raster files users need high throughput computing. Users can re-generate TEE-yearly and TEE-wave from TEE-daily on a laptop.
